# Extracting bioactive compounds and proteins from *Bacopa monnieri* using natural deep eutectic solvents

**DOI:** 10.1371/journal.pone.0300969

**Published:** 2024-03-29

**Authors:** Tan Phat Vo, Tran Ha Phuong Nguyen, Vy Khang Nguyen, Thi Cam Tu Dang, Le Gia Kiet Nguyen, Thanh Quynh Chung, Thi Thanh Huong Vo, Dinh Quan Nguyen

**Affiliations:** 1 Laboratory of Biofuel and Biomass Research, Faculty of Chemical Engineering, Ho Chi Minh City University of Technology (HCMUT), Ho Chi Minh City, Vietnam; 2 Vietnam National University Ho Chi Minh City, Thu Duc City, Ho Chi Minh City, Vietnam; Chaudhary Devi Lal University, INDIA

## Abstract

This study employed novel extraction methods with natural deep eutectic solvents (NADES) to extract bioactive compounds and proteins from *Bacopa monnieri* leaves. The conditional influence of ultrasonic-assisted extraction (UAE), microwave-assisted extraction (MAE), and enzymatic-assisted extraction (EAE) on the recovery efficiency of phenolics, proteins, flavonoids, and terpenoids was evaluated. The conditions of UAE were 50 mL/g LSR, 600W of ultrasonic power, and 30% water content with 40°C for 1 min to obtain the highest bioactive compounds and protein contents. The conditions of MAE were 40 mL/g LSR, 400W of microwave power with 30% water content for 3 min to reach the highest contents of biological compounds. The conditions of EAE were 30 mL/g of LSR, 20 U/g of enzyme concentration with L-Gly-Na molar ratio at 2:4:1, and 40% water content for 60 min to acquire the highest bioactive compound contents. Scanning electron microscopy (SEM) is employed to analyze the surface of *Bacopa monnieri* leaves before and after extraction. Comparing seven extraction methods was conducted to find the most favorable ones. The result showed that the UMEAE method was the most effective way to exploit the compounds. The study suggested that UMEAE effectively extracts phenolics, flavonoids, terpenoids, and protein from DBMP.

## 1. Introduction

*Bacopa monnieri* is a succulent herb belonging to the family Scrophulariaceae and commonly found in tropical regions. For thousands of years, *Bacopa monnieri* has been employed in India as a traditional herbal to enhance memory, alleviate neurological disorders, and reduce anxiety and stress. *Bacopa monnieri* contains 52 bioactive constituents, including several important compounds such as brahmine, herpestine, bacosides A and B, polyphenols, flavonoids, terpenoids, and proteins. This substance has antioxidants, antibacterial, antiinflammation, and anticancer activities, as well as treating cardiovascular diseases, protecting the liver, lowering blood sugar levels, and improving the immune system in the gut [1]. Therefore, this research has used scientific methods to extract biologically active compounds in *Bacopa Mennieri*.

The extraction procedure involves using solvents to separate bioactive components from materials. There are two categories of extraction techniques: conventional and innovative approaches. Conventional methods are simple operations; however, they have several drawbacks, such as long operation time, high energy consumption, and difficulty recovering bioactive substances. Innovative approaches like ultrasound-assisted extraction (UAE), microwave-assisted extraction (MAE), and enzyme-assisted extraction (EAE) have been developed to overcome these challenges. UAE is advantageous because it reduces extraction time, improves efficiency and quality, and uses less solvent. It works by using high-frequency sound waves to generate the cavitation effect, which improves the efficiency of the extraction process to obtain bioactive compounds from solid matrices. The ultrasonic-induced cavitation effect generates intense shear forces within the cavitation region, resulting in an elevated localized temperature and pressure, which causes cell wall degradation. This effect allows the substances inside the cell to be released into the surrounding liquid, facilitating the release of intracellular active components. Precise temperature control during the process is essential [2]. MAE is based on the absorption of microwave energy by polar extraction mediums, and then microwave energy is converted into thermal one based on oscillation, collisions, and friction of polar molecules in electromagnetic fields. As temperature increases, cell water evaporates, creating high pressure and rupturing the cell wall. As a result of this rupture, numerous pores are formed within cell walls, allowing extractants to penetrate more rapidly and improve the recovery of bioactive compounds [3]. EAE extracts compounds from plant cells using enzymes that break down cell wall components, containing cellulose, microfibrils, hemicellulose, pectin, and lignin. Several enzymes, including cellulase, hemicellulase, xylanase, and pectinase, are involved in this process [4].

Natural Deep Eutectic Solvents (NADESs) are formed by combining natural components, such as Hydrogen Bond Donors (HBD) and Hydrogen Bond Acceptors (HBA), found in various biological systems. The intermolecular hydrogen bonding between HBD and HBA contributes to the stability and physicochemical properties of NADESs. They have gained attention as alternative solvents due to their non-volatility, low toxicity, high biodegradability, and renewable precursors sourced from nature. NADESs have applications in green chemistry, extraction of bioactive compounds, pharmaceutical formulations, and enzymatic reactions. NADESs are efficient in extracting bioactive compounds. Currently, there has been extensive research conducted on the use of NADESs in bioactive compounds extraction from various sources such as plants and food processing waste, including *Abelmoschus sagittifolius* (Kurz) Merr, blueberry leaves, coffee husk waste. Previous research conducted by Tan Phat Vo et al. [5] focused on using NADES solvents to extract bioactive compounds from Bo Chinh ginseng. María Santos-Martín et al. [6] used NADESs-based UAE to extract phenolic compounds from blueberry leaves. The study discovered the optimal extraction conditions and used chromatographic methods to show that NADES-based UAE is more effective than traditional extraction methods. Askal Maimulyanti et al. [7] already utilized NADESs as an environmentally friendly option for phenolics extraction from coffee husk waste. The result showed that the most efficient combination of NADES solvent was choline chloride and proline ratio 1:1, leading to an extraction yield of 5.88 mg GAE/g and polyphenol concentration of 294.02 mg/L in the NADES solution. However, the effect of NADES-based single and combined extraction techniques on the recovery yield of phenolics, proteins, flavonoids, and terpenoids from *Bacopa monnieri* leaves was not elucidated.

Therefore, this study aimed to find a suitable green extraction technique to exploit phenolics, proteins, flavonoids, and terpenoids from *Bacopa monnieri*. The extractability of different NADES was compared to find the appropriate solvents before the influence of single extraction processes (UAE, MAE, and EAE) was examined. Subsequently, the combined extraction methods were conducted according to the proper conditions of single extraction methods. The comparison of NADES-based single and combined extraction approaches was conducted to find the most favorable extraction technique. Additionally, the variation in the surface of *Bacopa monnieri* leaves before and after extraction was also determined.

## 2. Materials and methods

### 2.1. Materials

*Bacopa monnieri* (producing area: Mekong Delta, Vietnam) was purchased from Thu Duc Agriculture Wholesale Market, Thu Duc City, Ho Chi Minh City, Vietnam. They were placed in a laboratory dryer HD-E804-2s for 48 hours (about two days) at a temperature of 45°C to allow water evaporation. Both the leaf and stem components were placed into a grinder and pulverized into a dried bacopa monnieri powder (DBMP). The resulting powder with the dried leaves and stems was then transferred into plastic containers.

### 2.2. Chemicals

Glycerin, D-glucose, lactic acid, and Trisodium Citrate Dihydrate were purchased from GHTECH Co. (Guangdong Guanghua, China). Choline chloride was purchased from Himedia Co. (Dindori, Nashik, India). L-(+)-tartaric acid and citric Acid Monohydrate were purchased from Xilong Scientific Co. (Shantou, Guangdong, China). Perchloric acid was purchased from Alpha Chemika Co. (Andheri West, Mumbai, Maharashtra, India). Vanilin was purchased from Shanghai Zhanyun Chemical Co. (Shanghai, Shanghai, China). Folin’s reagent (purity ≥ 1.9N), 6-hydroxy-2,5,7,8-tetramethylchroman-2-carboxylic acid, aluminum chloride, potassium acetate, ethanol, sodium dihydrogen phosphate dihydrate, salicylic acid, 2,2’-azino-bis (3-ethylbenzothiazoline-6-sulfonic acid), 2,2-diphenyl-1-picrylhydrazyl, disodium hydrogen phosphate dodecahydrate, starch, and cellulast 1.5L were purchase from Sigma Chemical Co. (St. Louis, MO).

### 2.3. NADES production and screening

Eight different NADES were selected to evaluate their phenolic, flavonoid, and terpenoid extractability from DBMP. The combination of three HBD, namely citric acid, tartaric acid, and lactic acid, and HBA (glycerol, glucose, and choline chloride) with a proper 2:1 molar ratio is shown in Table 1. The mixture of HBA and HBD was heated at 90°C until the appearance of a transparent liquid. NADES were successfully synthesized when they did not crystallize at ambient temperature.

**Table 1:** Eight NADES were prepared for this research.

No.	HBD	HBA	Abbreviation	Molar ratio	Water content (wt.%)
1	Lactic acid	D-glucose	L-Glu	2:1	20%
2		Glycerol	L-Gly	2:1	
3		Choline chloride	L-Cho	2:1	
4	Citric acid	D-glucose	Ci-Glu	2:1	
5		Glycerol	Ci-Gly	2:1	
6		Choline chloride	Ci-Cho	2:1	
7	Tartaric acid	Glycerol	T-Gly	2:1	
8		Choline chloride	T-Cho	2:1	

### 2.4. Ultrasound-assisted, microwave-assisted and Enzyme-Assisted Extraction

#### 2.4.1. Ultrasound-assisted extraction

The determined quantity of DBMP was combined with 10 mL of NADES in an amber glass container and subjected to an ultrasonic bath (RS22L 40 kHz, Rama Viet Nam Joint Stock Company, Ho Chi Minh City, Vietnam). The experiment involved various (LSR, spanning from 10 to 60 mL/g), ultrasonic power (ranging from 0 to 900 W in increments of 150 W), water contents (ranging from 10% to 50%, g/g), and a temperature range of 30 to 70°C with 10°C intervals for various extraction durations (5, 10, 15, 20, and 25 minutes).

#### 2.4.2. Microwave-assisted extraction

A precise quantity of DBMP was blended with 10 mL of NADES in an amber glass vessel. The mixture was exposed to microwave irradiation (model R-205VN 2450 MHz, Sharp Corporation, Japan). The experiment encompassed varying LSR ranging from 10 to 60 mL/g, water contents (ranging from 10% to 50%, g/g), and distinctive microwave power settings, including 0, 240 W, 400 W, and 800 W for varying durations of 0.5, 1, 2, 3, and 4 minutes.

#### 2.4.3. Enzyme-Assisted Extraction

DBMP was integrated with NADES with different LSR (10, 20, 30, 40, 50, and 60 mL/g) and water content within the range of (20, 30, 40, and 50%, g/g). After, cellulase enzyme was introduced at diverse concentrations ranging (from 0 to 30 U, with an interval of 5 U). Then, a water bath was used to incubate the mixture at 55°C (Model: SBS40 24L, Stuart, England) for different incubation periods (30, 60, 90, 120, and 150 minutes).

#### 2.4.4. Combination of three methods

The four combined extraction processes were conducted. The experimental conditions of a single method used in the combined extraction process were selected from the results of sections 2.4.1, 2.4.2, and 2.4.3. A determined quantity of DBMP and 10 ml of NADES were combined in an amber glass, and then the mixture underwent treatment by the four combined extraction processes. Ultrasonic-Microwave-Assisted Extraction (UMAE): The mixture underwent ultrasonic treatment before being subjected to a microwave oven. Ultrasonic-Enzyme-Assisted Extraction (UEAE): The mixture underwent treatment using an ultrasonic bath before Cellulast 1.5L was introduced to the extraction medium. Microwave-Enzyme-Assisted Extraction (MEAE): the mixture underwent microwave treatment before Cellulast 1.5L was introduced to the extraction medium. Ultrasonic Microwave-Enzyme-Assisted Extraction (UMEAE): Ultrasonic treatment was first applied to the mixture, then the mixture was subjected to a microwave oven; subsequently, the mixture underwent the hydrolysis process using Cellulast 1.5L in the water bath.

### 2.5. Bioactive compounds and soluble proteins determination

#### 2.5.1. Total phenolic content (TPC)

TPC was quantified using Wu et al. [8] with minor changes. The samples (0.25 ml) were added to test tubes before 0.25 ml of Folin’s phenol reagent (0.19N) was added. After that, the sample was incubated for 5 min before Na_2_CO_3_ (0.5 ml, 7.5%) and distilled water (4 ml) was added. The sample absorbance was measured at 765nm using a colorimetric method with UV visible spectrophotometers (Model: UV1200, Yoke Instrument Co., Ltd, China). TPC was presented as milligrams of gallic acid equivalent per gram of dried weight (mg GAE/g).

#### 2.5.2. Total flavonoid content (TFC)

TFC was assayed using Wu et al. [8] with minor modifications. The samples (0.5 ml) were added to the test tubes before ethanol (1 ml, 96°) was placed. After that, potassium acetate solution (0.1 ml, 1M), aluminum trichloride solution (0.1 ml, 10%), and 3.3 ml of distilled water was added before the samples were in the dark place for 30 min. The absorbance of samples was obtained at 415nm using a colorimetric method. A unit of TPC was milligrams of Rutin acid equivalent per gram of dried basis weight (mg RE/g).

#### 2.5.3. Total terpenoid content (TTC)

TTC was measured by the Biswas method with minor changes [9]. The samples were combined with perchloric acid (1 ml, 70% volume/volume, % v/v) and vanillin in acetic acid (0.3 ml, 5% g/ml). Afterward, the mixture was put in the water bath (Model: SBS40 24L, Stuart, England) at 60°C for 45 minutes. Acetic acid was dropped in the test tubes before the absorbance of the mixture was read at 548 nm using a colorimetric method. A unit of TTC was milligrams of ursolic acid equivalent per gram of dried basis weight (mg UE/g).

#### 2.5.4. Total Protein Content (TPRC)

The Bradford reagent was prepared according to the formula: 0.05g Coomassie Blue, 50ml ethanol, and 150ml concentrated phosphoric acid. The mixture was placed and filled up to 1000ml in a volumetric flask before it was filtered through filter papers under vacuum pressure. The initial sample (0.25 mL) was transferred into a tube before introducing the Bradford reagent (3mL). The blend was left in a dark site for 5 min. Subsequently, the absorbance of the mixture was assessed at a wavelength of 595 nm using a colorimetric method. TPRC values are quantified in milligrams of bovine serum equivalent per gram of dry weight (mg BSAE/g) [10].

### 2.6. Antioxidant activity determination

The Lingfeng Wu method, with a slight modification, was employed to quantify DPPH [8]. In this procedure, 0.5 ml of the samples was mixed with 3.5 ml of a DPPH solution (100 μM in absolute ethanol) and incubated in darkness for 30 minutes at 30°C. The sample absorbance was read at 515 nm employing a UV-vis spectrophotometer. The method outlined by Pattrathip Rodsamran for ABTS determination was followed [11]. ABTS solution (25 ml, 7.4mM) and potassium persulfate (25 ml, 2.45 mM) were reacted for 16 hours at ambient temperature in the dark to generate ABTS^+^. The resulting ABTS+ solution was obtained by diluting it with distilled water to achieve an absorbance of 1.0 at 734 nm. To assess ABTS scavenging activity, 0.1 ml of samples were mixed with 3.9 ml of ABTS+, followed by a 20-minute incubation in darkness, and the absorbance was measured at 734 nm. Hydroxyl radical scavenging capacity (OH) was quantified by the Lingfeng Wu method [8]. The results for DPPH, ABTS, and OH scavenging activities were expressed as micromoles of Trolox equivalent per gram of dried materials (μM TE/g).

### 2.7. Surface morphology of DBMP

Scanning electron microscopy (model: Prisma E SEM, Thermo Fisher Scientific, Waltham, Massachusetts) was used to identify the surface changes of DBMP before and after treatment using different extraction methods. Sample preparation followed the Patil method [12], where samples were placed on carbon tape to prevent any loss and enclosed on the sample plate. The samples were then coated with a layer of gold metal under vacuum conditions. The samples were examined directly under SEM at varying magnifications and 5 kV.

### 2.8. Statistical analysis

The experiments were replicated in triplicate, and the results were represented as the mean ± standard deviation (SD). Statistical analysis was carried out using Minitab 19 (Minitab, Inc, USA), which included analysis of variance (ANOVA) with a confidence level of 95%. Graphical representations were generated using Origin Pro software (Origin Lab, USA).

## 3. Results and discussions

### 3.1. Evaluating the Extraction Performance of NADES-based UAE

**Fig 1:**
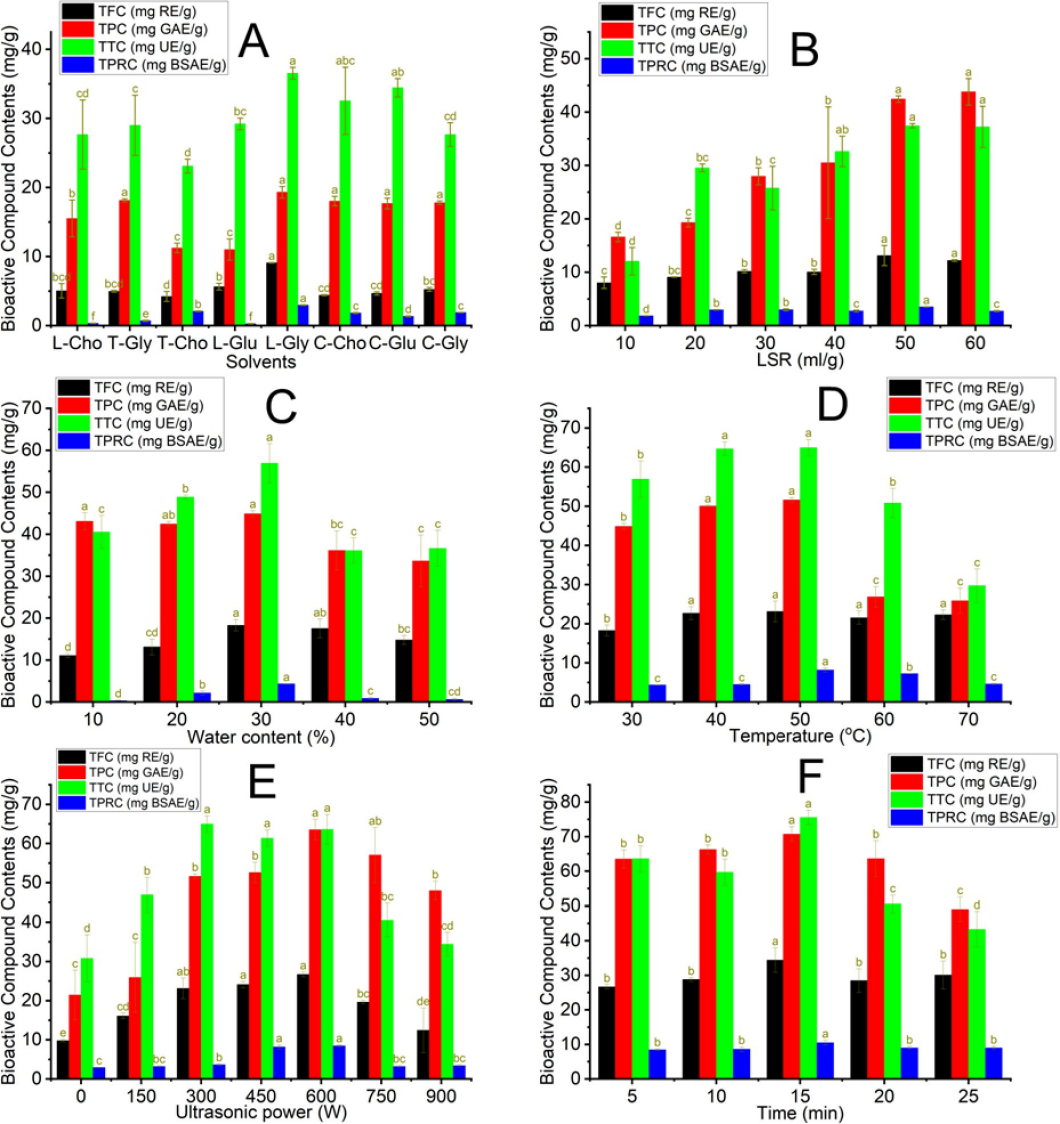
The impact of NADES-based UAE parameters on the recovery efficiency of phenolics, flavonoids, terpenoids, and proteins; (A): the effect of solvent at LSR 20 ml/g, water contents 20%, temperature 30°C, ultrasonic power 300W and time 5 minutes; (B): the effect of LSR at solvent L-GLy, water contents 20%, temperature 30°C, ultrasonic power 300W and time 5 minute; (C): the effect of water contents at L-Gly, LSR 50 ml/g, temperature 30°C, ultrasonic power 300W and time 5 minute; (D): the effect of temperature at solvent L-Gly, LSR 50 ml/g, water contents 30%, ultrasonic power 300W and time 5 minute; (E): the effect of ultrasonic power at solvent L-Gly, LSR 50 ml/g, water contents 30%, temperature 50°C and time 5 minute; (F): the effect of time at solvent L-Gly, LSR 50 ml/g, water contents 30%, temperature 50°C and ultrasonic power 600W. Different characters (a, b, c, d, f, and e) show the significance of statistical analysis.

NADES compositions have a critical role in the extraction performance of bioactive compounds from DBMP because they contribute to solvent properties such as viscosity, solvation, and polarity [13]. Eight NADES were synthesized from organic acids as HBD and three HBD (glucose, glycerol, and choline chloride) with 20% water content. TPC, TFC, TTC, and TPRC were employed to evaluate the efficiency of NADES-based UAE. The effect of NADES types on the UAE process was evaluated at ultrasonic power 300W, temperature 30°C, 20% water content, and 5 min of extraction time. The results (Fig 1A) showed that L-Gly exhibited the highest extraction effectiveness for both flavonoids, phenolics, terpenoids, and proteins at 9.10±0.05 mg RE/g, 19.31±0.83 mg GAE/g, 36.54±0.84 mg UE/g, 2.97±0.04 mg BSAE/g, respectively. The solubility of bioactive compounds is based on the principle of "like dissolves like" [14]. The high extraction yield of L-Gly can be attributed to the low viscosity and the polar similarity between it and bioactive compounds [15–17]. In contrast, T-Cho exhibited the lowest TFC, TPC, TTC, and TPRC values at 5.36±1.05 mg RE/g, 15.51±2.67 mg GAE/g, 27.65±5.00 mg UE/g, 0.34±0.04 mg BSAE/g, respectively. The solubility of bioactive compounds in T-Cho is lower compared to L-Gly due to tartaric acid being more polar than lactic acid. Moreover, L-Gly also indicated the highest protein extraction at 2.97±0.04 mg BSAE/g. It can be attributable to amino and carboxyl groups forming hydrogen bonds with carboxyl groups of lactic acid and hydroxyl groups of glycerine, improving protein hydration and increasing solubility [18]. This effect increases the extraction efficiency of protein. On the contrary, the lowest TPRC extraction efficiency of L-Cho and L-Glu at 0.34±0.038 mg BSAE/g and 0.28±0.05 mg BSAE/g, respectively, were attributed to the lower polarity of amino acid in DBMP proteins, resulting in the decreased solubility of proteins in L-Cho and L-Glu. Tan Phat Vo et al. presented the influence of polar solvents on bioactive compounds’ extraction performance from *Garcinia mangostana* L [19]. The study showed that Lactic acid and 1,2-Propanediol achieved the highest flavonoids and phenolics extraction yield. Therefore, L-Gly was selected as the solvent for the extraction procedures in this study.

### 3.2. Effect of ultrasonic-assisted extraction conditions

The extraction yield of bioactive compounds from DBMP is affected by numerous parameters, such as LSR, water content, ultrasonic power, temperature, and time. LSR, water content, and temperature can impact the properties of solvents, such as viscosity, while temperature and time affect the running costs and the durability of bioactive compounds during the UAE process [19]. The viscosity plays a vital role in deciding the traveling and intensity of ultrasound in the extraction medium; thus, finding suitable parameters for the UAE process is necessary. The results of effect of solvent types and UAE conditions on the recovery of bioactive compounds and protein are presented in Fig 1A–1F.

#### 3.2.1 Effect of liquid-to-solid ratios in the NADES-based UAE process

This research studied the effect of different LSR (10, 20, 30, 40, 50, and 60 mL/g) on the extraction yield of flavonoids, phenolic, terpenoids, and protein using NADES-based UAE conditions. As demonstrated in Fig 1B, TFC, TPC, TTC, and TPRC increased gradually from 10 to 60 mL/g and peaked at an LSR of 50 mL/g. The increase in LSR helps enhance the mass transfer due to the significant differences in solute concentration between materials and L-Gly. A decrease in the viscosity of the extraction medium at higher LSR can result in the shrinkage of the cavitation threshold, which is the minimum acoustic pressure required to initiate the formation of cavities during rarefaction cycles [19]. However, as LSR increased over 50 mL/g, TPRC decreased. The viscosity of L-Gly experiences a continuous decline with the augmentation of LSR surpassing 50 mL/g. This effect can heighten the extraction medium’s ultrasonic energy acquisition, leading to protein denaturation and a reduction of protein compound content in L-Gly. Rodrigues et al. [20] observed that the amount of phenolics obtained from coconut (*Cocos nucifera*) shell powder increased when LSR ranged from 20:1 to 50:1 ml/g. Therefore, the results reported that in LSR 50 mL/g, TFC, TPC, TTC, and TPRC were the highest at 13.12±1.89 mg RE/g, 42.43±0.61 mg GAE/g, 37.45±0.41 mg UE/g, and 3.51±0.03 mg BSAE/g, when LSR was 50 mL/g.

#### 3.2.2. Effect of water content in the NADES-based UAE process

The impact of the water content (10–50%) of L-Gly on the yield of the extraction process was assessed. The NADES-based UAE parameters were kept at 50 mL/g, 300 W, and 30°C for 5 min. It can be observed from Fig 1C that TFC, TPC, TTC, and TPRC gradually increased as the water content in L-Gly rose from 10 to 30%. Regarding protein extraction, the water content of 10% is insufficient for protein hydration during the extraction process, leading to a low extraction yield of soluble protein [21]. Increasing water can lower the viscosity, density, and intramolecular interactions of NADES [22]. This effect increases molecular motion and the number of hydrogen bonds among NADES, water, and solute. These networks assist in improving surface tension, which enhances the contact area between L-Gly and DBMP, improving the extraction efficiency of proteins and bioactive compounds. However, 50% of water content in L-Gly can raise L-Gly polarity and disrupt the intra-hydrogen bond network of NADES. This effect reduces the L-Gly solubility, declining the extraction yield of proteins and bioactive compounds [23]. M. Guzmán-Lorite et al. used NADES to recover proteins from pomegranate seeds [21]. The research found that the optimal water content was 35% (v/v), and the optimal protein content was 13.3g of proteins/100g of dried defatted seeds. Thus, it was essential for L-Gly to contain a water content of 30% to achieve the maximum extraction efficiency of TFC, TPC, TTC, and TPRC from DBMP with 18.27±1.38 mg RE/g, 44.87±0.76 mg GAE/g, 56.92±4.64 mg UE/g, and 4.34±0.04 mg BSAE/g, respectively.

#### 3.2.3. Effect of temperature in the NADES-based UAE process

The impact of temperature on the extraction of bioactive compounds using L-Gly was studied across a temperature range of 30–70°C, and the outcomes of experiments are presented in Fig 1D. The outcomes showed that TPC, TTC, TFC, and TPRC were the highest at 40°C and 50°C. In contrast, TPC, TTC, TFC, and TPRC decreased by 1.9, 1.3, and 1.1 times at 60°C, respectively, compared to those at 50°C. Increasing the temperature enhances the solubility of these bioactive compounds, leading to higher extraction yields. However, decreasing surface tension and cavitation bubble size at higher temperatures can trigger a decline in the intensity of rupture bubbles. As a result, the transfer of bioactive compounds from DBMP to L-Gly is hindered. Moreover, high temperatures could cause the destruction of bioactive compounds, resulting in a lower extraction yield [24]. Additionally, the structure of proteins can be affected by heating, leading to increased hydrophobicity of the protein surface. This effect can be the primary reason for the unfolding and separating of protein subunits, which causes protein precipitation, reducing extraction efficiency [25, 26]. Hao Huang et al. reported increased crocins of gardenia fruits when extraction temperature increased. The result showed that crocins peaked at the highest extraction temperature, about 35°C [27]. Hence, the favorable temperature for extracting bioactive compounds and proteins was found to be 50°C, in which TPC, TTC, TFC, and TPRC were 51.62±0.65 mg GAE/g, 64.97±2.10 mg UE/g, 23.12±2.63 mg RE/g, and 8.15±0.54 mg BSAE/g, respectively.

#### 3.2.4. Effect of ultrasonic power in the NADES-based UAE process

An exploration of the ultrasonic power effect on the extracting efficacy of phenolics, terpenoids, flavonoids, and proteins was conducted from 0 to 900W. As depicted in Fig 1E, it can be observed that the values of TFC, TPC, TTC, and TPRC increase as the power increases, but they start to decrease when the power continues to rise. The highest TFC, TPC, TTC, and TPRC were at 600 W. It can be ascribed to the heightened disruptions of cell walls and micropore generation within cellular tissues, resulting from high shear forces, fragmentation, and localized pressures induced by acoustic cavitation. This phenomenon enhances the yield of extraction and diffusivity. However, as ultrasonic power continues increasing to 750 W, the quantity of cavitation bubbles also rises, leading to significant inter-bubble collisions, deformations, and non-spherical collapses. This phenomenon can diminish the impact of bubble implosion. Furthermore, the intense localized temperatures and the generation of high levels of free radicals during bubble implosion can contribute to the degradation of bioactive compounds, reducing the extraction yield [28, 29]. Guanghui Chen et al. noted similar results in the process of extracting essential oil from chickpeas. The experimental results demonstrated that when the ultrasound power was under 300 W, there was a consistent rise in the extraction yield as the ultrasound power increased. Nevertheless, the extraction yield starts to decline when surpassing 300W [29]. Accordingly, an ultrasonic power of 600 W was appropriate for NADES-based UAE to achieve the values of TFC, TPC, TTC, and TPRC, which were 26.68±0.60 mg RE/g, 63.53±2.64 mg GAE/g, and 63.58±3.82 mg UE/g, 8.46±0.25 mg BSAE/g, respectively.

#### 3.2.5. Effect of time in the NADES-based UAE process

This research studied the impact of time from 5 to 25 min under the NADES-based UAE, and the other conditions were kept at 600 W of ultrasonic power, 30% water content, 40°C, and 50 mL/g LSR. As illustrated in Fig 1F, the TPC, TFC, TTC, and TPRC of the DBMP extracts showed an increase in extraction time, from 5 min to 15 min, and reached a peak at 15 min. However, as the extraction time exceeded 15 min, bioactive compounds exhibited a gradual decline. The reason for the increase in the compound content was believed to promote the yield of flavonoids, phenolics, terpenoids, and proteins when extraction time was extended. On the contrary, the continuously extended extraction time of NADES-Based UAE over 15 min can lead to the excessive absorption of ultrasonic power, degrading phenolics, flavonoids, and terpenoids [30]. Hao Huang et al. reported increased crocins of gardenia fruits when extraction temperature increased. The result showed that crocins peaked at the highest extraction temperature, about 35°C [27]. In conclusion, flavonoids, phenolics, terpenoids, and proteins peaked at 15 min with 34.41±3.55 mg RE/g, 70.73±2.18 mg GAE/g, 75.53±2.10 mg UE/g; 10.47±0.69 mg BSAE/g, respectively.

### 3.3. Effect of microwave-assisted extraction conditions

**Fig 2:**
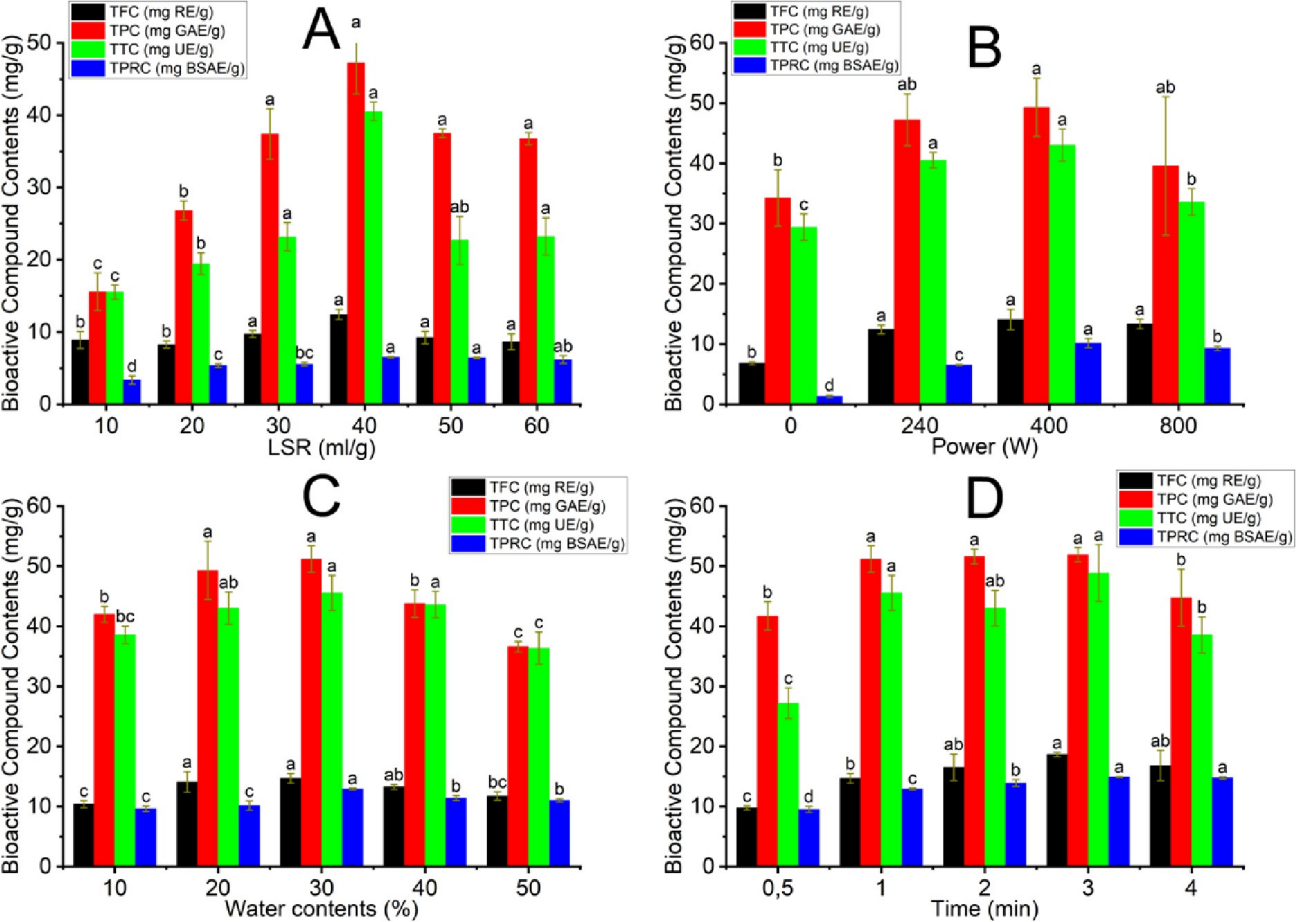
The impact of NADES-based MAE parameters on the recovery efficiency of phenolics, flavonoids, terpenoids, and proteins; (A) the effect of LSR at power 240W, water contents 20% and time 1 minute; (B) the effect of power at LSR 40 ml/g, water contents 20% and time 1 minute; (C): the effect of water contents at LSR 40 ml/g, power 400W and time 1 minute; (D): the effect of time at LSR 40ml/g, power 400W and water contents 30%. Different characters (a, b, c, d, and e) show the significance of statistical analysis.

The extraction yield of bioactive compounds from DBMP is affected by numerous parameters, such as LSR, water content, microwave power, and time. LSR, water content, and microwave power can impact the properties of solvents, such as viscosity and dielectric constant, while microwave power and time affect the running costs and the durability of bioactive compounds during the MAE process. Viscosity plays a vital role in deciding the release rate and mass transfer of solute into the extraction medium; thus,, finding suitable parameters for the MAE process is essential [31]. The effect of MAE conditions on the recovery of bioactive compounds and proteins is showed in Fig 2A–2D.

#### 3.3.1. Effect of liquid-to-solid ratios in the NADES-based MAE process

The research investigated the effect of different LSR (10, 20, 30, 40, 50, and 60 mL/g) on the extraction yield of flavonoids, phenolic, terpenoids, and protein using NADES-based MAE conditions. The other conditions were held at a microwave power of 240 W and water content of 20% for 1 min. As demonstrated in Fig 2A, TFC, TTC, TPC, and TPRC reached a high at 40 mL/g. Increasing LSR can lower the overall viscosity of the extraction medium and establish a greater contact between the solvent and solute. This effect ensures the even exposure of substances and microwave irradiation and the greater capacity of microwave energy absorption, increasing the heating efficiency of the microwave. Improving the microwave-based heating effect increases the mass transfer and solubility of flavonoids, phenolics, terpenoids, and proteins, enhancing their extraction yield [32]. Furthermore, protein can undergo denaturation when the LSR is decreased. When LSR increased over 40 mL/g, TFC, TTC, TPC, and TPRC contents were reduced. Since the LSR increased with the greater volume of L-Gly, the solvent and solute can receive more energy from the microwave. This effect can destroy flavonoids, terpenoids, phenolic, and protein content [33]. Marija Ranić et al. estimated the effect of LSR on TPC extraction from spent espresso coffee grounds [34]. The highest total extract yield was recorded when LSR was 12 mL/g. Hence, TFC, TTC, TPC, and TPRC were the highest at 40 mL/g, which was 12.44±0.73 mg RE/g of TFC, 47.25±4.30 mg GAE/g of TPC, 40.53±1.27 mg UE/g of TTC and 6.53±0.09 mg BSAE/g of TPRC.

#### 3.3.2. Effect of microwave power in the NADES-based MAE process

An investigation of the effect of microwave power (0, 240, 400, and 800 W) on the bioactive compound and protein extraction yield was conducted at 40 mL/g LSR and 20% water content of L-Gly in 1 min. The results, as depicted in Fig 2B, revealed an improvement in the extraction efficiency of bioactive compounds and proteins when microwave power was set between 0 and 400 W. Increasing microwave power leads to an increase in systemic temperature and pressure, causing plant matrix destruction, also the diffusion and dissolution of solute. Therefore, the diffusivity of bioactive compounds and proteins into NADESs is enhanced, resulting in higher extraction yield. On the contrary, increasing microwave power to 800 W caused a decrease in the TPC, TTC, and TPRC while TFC was stable. High microwave power can cause TPC, TTC, and TPRC to decline in DBMP extracts because of the degradation of thermally sensitive phenolic and terpenoid compounds and protein denaturation [35]. The results showed a similar trend to those of Shinta R. Dewi et al., who recovered phenolics from cacao pod husk using MAE [36]. Therefore, microwave power at 400 W was appropriate for NADES-based MAE to achieve the highest TPC, TFC, TTC, and TPRC from DBMP at 49.32±4.83 mg GAE/g, 14.11±1.71 mg RE/g, 43.03±2.68 mg UE/g and 10.17±0.78 mg BSAE/g, respectively.

#### 3.3.3. Effect of water content in the NADES-based MAE process

The effect of the water content in a wide range from 10 to 50% was evaluated through the NADES-based MAE. The other conditions were held at a microwave power of 400 W and LSR of 40 ml/g for 1 min. The extraction yield of all bioactive compounds into NADES increases as the water content from 10 to 30%, which depicts the most extractable efficiency. The dielectric constant refers to a solvent’s capacity to absorb microwave energy and experience polarization. Dielectric loss measures the efficacy of converting electromagnetic energy into thermal energy. The association order of water molecular structure greatly resists the thermal motions, thus contributing to water’s substantial dielectric constant [37]. Despite its super dielectric loss properties compared to alternative solvents, water also speeds up overheating, accumulating much pressure based on its high dipole moment. This effect results in the intensified destruction of plant cell walls, increasing the number of pores on the material surface. Numerous pores improve solvent permeability and release the target analyte from the material to NADES, thereby amplifying the extraction performance of bioactive components [38]. The water added into NADES at higher concentrations of 40 and 50%, TPRC experienced a decline. The addition of water exceeding 30% decreased the extraction yield due to the probable disintegration of the supramolecular formation of NADEs. The results had a similar trend to our previous study, which extracted terpenoids and phenolics in *Abelmoschus sagittifolius* (Kurz) Merr roots using NADES-based MAE [5]. Therefore, it recommends conducting the NADES-based MAE process with a water content in L-Gly of 30% for maximum efficiency, which was indicated by the highest at 51.20±2.19 mg GAE/g TPC, 14.71±0.80 mg RE/g TFC, 45.53±2.93 mg UE/g TTC, 12.93±0.16 mg BSAE/g TPRC.

#### 3.3.4. Effect of time in the NADES-based MAE process

The impact of microwave irradiation durations (0.5, 1, 2, 3, and 4 minutes) on the acquisition of phenolic compounds, terpenoids, flavonoids, and proteins was investigated under conditions of 40 mL/g liquid-to-solid ratio (LSR), 30% water content in the extraction medium (L-Gly), and 400 W microwave power. As illustrated in Fig 2D, the highest values for TFC, TPC, TTC, and TPRC were observed at 3 min. It has been noticed that the yield of extraction tends to rise as the extraction duration is extended. However, the extraction performance of phenolics and terpenoids decreased as the extraction time was further extended [35]. Lengthening the exposure to microwave irradiation leads to higher temperatures and the accumulation of pressure, causing greater destruction of plant cell tissue. The ruptured plant cells lead to heightened swelling and greater contact surface area between solvents and solutes, amplifying the extraction yield [39]. As Xuejun Pan et al., the findings suggest that as the MAE time increased, there was an augmentation in the extraction of polyphenols. The peak of this enhancement was observed at 4 minutes of MAE. However, the MAE time exceeded 4 minutes, and the extraction of caffeine declined as the duration increased [40]. Hence, a microwave irradiation duration of 3 minutes proved suitable for achieving the highest TFC, TPC, TTC, and TPRC from DBMP at 18.65±0.35 mg RE/g, 51.90±1.19 mg GAE/g, 48.86±4.74 mg UE/g, and 14.90±0.08 mg BSAE/g, respectively.

### 3.4. Effect of Enzymatic-assisted extraction conditions

**Fig 3:**
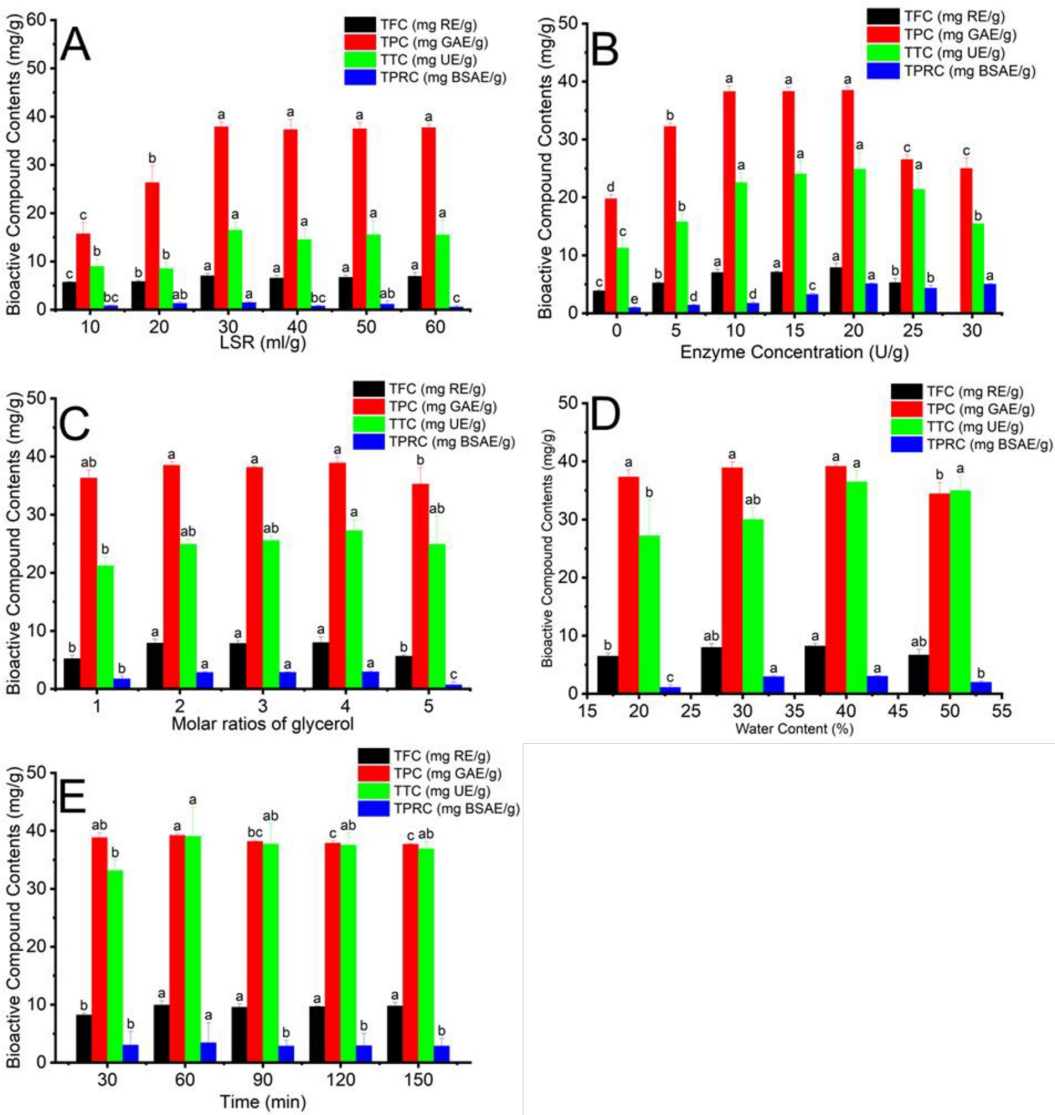
The impact of NADES-based EAE parameters on the recovery efficiency of phenolics, flavonoids, terpenoids, and proteins; (A): the effect of LSR at enzyme concentration 10 U/g, molar ratios of glycerol 2, water content 30% and time 30 minutes; (B): the effect of enzyme concentration at LSR 30 ml/g, molar ratios of glycerol 2, water content 30% and time 30 minutes; (C): the effect of molar ratios of glycerol at LSR 30 ml/g, enzyme concentration 20 U/g, water content 30% and time 30 minutes; (D): the effect of water content at LSR 30 ml/g, enzyme concentration 20 U/g, molar ratios of glycerol: 4 and time 30 minutes; (E): the effect of time at LSR 30 ml/g, enzyme concentration 20 U/g, molar ratios of glycerol: 4 and water content 40%. Different characters (a, b, c, d, and e) show the significance of statistical analysis.

The extraction yield of bioactive compounds from DBMP is affected by numerous conditions, such as LSR, water content, enzyme concentration, the molar ratios of components, and time. LSR, water content, and the molar ratios of components can impact the properties of solvents, such as viscosity and the melting temperature of solvents, while time can affect the running costs and the durability of bioactive compounds during the EAE process. The melting temperature of HBD and HBA can affect the final melting temperature of solvents. The lower melting point of HBA and HBD leading that of NADES and vice versa. The sodium ion acts as a pH adjuster to ensure a suitable pH for maintaining cellulase activities. The viscosity is vital in deciding the release rate and mass transfer of solute into the extraction medium [41]. Enzyme concentration influences the recovery yield of bioactive compounds by changing the cell wall-enzyme interaction [42]. Therefore, investigating suitable parameters for the UAE process is essential [31]. The effect of EAE conditions is illustrated in Fig 3A–3E.

#### 3.4.1. Effect of liquid-to-solid ratios in the NADES-based EAE process

The effect of different LSR in the NADES-Based EAE (10, 20, 30, 40, 50, and 60 mL/g) was implemented at 30% water content, 10 U/g enzyme concentration for 30 min. As demonstrated in Fig 3A, flavonoids, phenolic, terpenoids, and protein content increased gradually to 30 mL/g and peaked at this LSR. When LSR exceeded 30 mL/g, LSR, TFC, TPC, and TTC did not change, while protein content decreased after 30 mL/g. Ning Wang and Qian Li (2022) also studied the effect of LSR in the NADEs combined with ultrasound-assisted enzymolysis [43]. This research result showed that bioactive compounds reached the highest concentration after extraction under 30 mL/g LSR. Increasing LSR in the extraction of biologically active compounds can promote the contact of material and cellulase, improving cell wall breakage. However, flavonoid, phenolic, and terpenoid contents did not significantly change when the volume of NADES was increased. In conclusion, flavonoids, phenolic, terpenoids, and protein contents were the highest at 30 mL/g LSR in the NADES-Based EAE Process at 29.98±2.08 mg RE/g TFC, 37.91±0.96 mg GAE/g TPC, 16.47±1.73 mg UE/g TTC, 1.46±0.24 mg BSAE/g TPRC.

#### 3.4.2. Effect of enzyme concentration in the NADES-based EAE process

Enzyme concentration (0, 5, 10, 15, 20, 25, and 30 U/g) was analyzed at 30 mL/g LSR, 30% water content of L-Gly and 2:1:1 L-Gly-Na molar ratio for 30 min to evaluate bioactive compounds and protein extraction yield. Fig 3B shows that the highest levels of TPC, TFC, and TTC were observed when enzyme concentration ranged from 10 to 20 U/g. Additionally, the greatest protein extraction yield was obtained at 20 U/g. Cellulase activity can be maintained in NADES because it forms hydrogen bonds with the active site of cellulase (Asp252 and Asp392). Increasing enzyme concentration improves the number of hydrogen bonds in the extraction media, leading to the hydrolysis of cellulose enhancement. This effect results in the breakdown of cell walls, improving phenolics, flavonoids, terpenoids, and protein release [44, 45]. On the contrary, bioactive compounds and protein extraction efficiency declined when exceeding enzyme concentration was over 20 U/g. Excessive enzyme concentrations can produce undesirable product production during extraction [44]. Research conducted by Zhang et al. [46] optimized the extraction of oil from bayberry (Myrica rubra) kernels using an aqueous enzymatic method. The result showed that under optimum enzyme concentration of 3.17% (w/w), the oil extraction yield was 31.5%. Therefore, the appropriate enzyme concentration for extracting bioactive compounds and proteins from DBMP was determined to be 20 U/g, in which TFC, TPC, TTC, and TPRC were 7.91±0.63 mg RE/g, 38.50±0.58 mg GAE/g, 24.90±0.87 mg UE/g, and 5.10±0.16 mg BSAE/g.

#### 3.4.3. Effect of molar ratios of glycerol in the NADES-based EAE proces

Molar ratios can exert a profound influence on the physicochemical properties of NADES, which plays a pivotal role in determining the extraction yield of phenolics, flavonoids, terpenoids, and proteins. The highest result in extracting both bioactive compounds and proteins was observed in Fig 3C with a L-Gly-Na molar ratio of 2:4:1. Increasing glycerol molar ratios can increase hydrogen bonds between HBA (glycerol) and HBD (sodium citrate, and lactic acid), thereby enhancing the solubility of bioactive compounds and proteins in solvents and leading to higher extraction yield [47]. However, the extraction efficiency of phenolics, flavonoids, terpenoids, and proteins declined when increasing molar ratios of L-Gly-Na up to 2:5:1 [44]. The solubilization of residual glycerol can decrease the quantity of hydrogen bonding sites interacting with cellulase. This phenomenon can reduce the diffusion and solubility of cellulase in NADES, decreasing the enzyme stability. The reduction in enzyme stability leads to reduced enzyme activity, decreasing cell wall degradation capacity and extraction yield [48]. This trend was reported by Suman Kumar Saha et al., who extracted polyphenols from *Aegle marmelos* using NADES-based UAE [48]. Therefore, the L-Gly-Na molar ratio at 2:4:1 was an appropriate media for cellulose hydrolysis using cellulase at 8.00±0.98 mg RE/g TFC, 38.93±1.01 mg GAE/g TPC, 27.26±1.91 mg UE/g TTC, 2.96±0.20 mg BSAE/g TPRC.

#### 3.4.4. Effect of water content in the NADES-based EAE process

Within the EAE process, all given bioactive compounds in DBMP were used to assess the impact of water content ranging from 20 to 50% on extraction yield. The parameters were kept at an LSR of 30 mL/g and a molar ratio of L-Gly-Na at 2:2:1 for 30 min. As shown in Fig 3D, TPC remained stable, whereas TFC, TTC, and TPRC peaked at 40% of water content in L-Gly-Na. The augmentation in the quantity of intra-main chains hydrogen bonds facilitated by the introduction of water grows the interaction between NADES and enzymes. The stability of the enzyme’s secondary and tertiary structure is attained within the NADES solvent as a result of the establishment of hydrogen bonds among amino acids, particularly at the enzyme’s active site along protein chains [47]. The slight decrease in outcome occurred at 50%, similar to the reasons for sections 3.2.2 and 3.3.3. F. Ruiz-Terán et al. investigated the impact of enzymatic extraction on the amount of average vanillin obtained from 50 g of Vanilla Pods by distinct combination, especially the utilization of 15 mL of cellulase and 135 mL of water [49]. After 8 hours of reaction, the outcome revealed a high yield of nearly 1,17g/mg of extracted vanillin content. Hence, 40% is recommended as the most effective water ratio employed in NADES preparation, which obtained the highest results of TFC, TPC, TTC, and TPRC, corresponding to 8.23±0.29 mg RE/g, 39.17 ±0.36 mg GAE/g, 36.46±1.95 mg UE/g, 3.02±0.07 mg BSAE/g.

#### 3.4.5. Effect of time in the NADES-based EAE process

The effects of varying extraction times (30–180 minutes) on the extraction efficiency of flavonoids, phenolics, terpenoids, and proteins were examined under conditions of 30mL/g liquid-to-solid ratio (LSR), 40% water content in the extraction medium (L-Gly-Na), 20 U/g enzyme concentration and 4 mol molar ratios of glycerol. As shown in Fig 3E, the highest values of TFC, TPC, TTC, and TPRC were observed at 60 minutes, followed by ream. The graph showed that the trend increased until 60 min and remained stable. This outcome could be attributed to the progressive cellulose degradation by cellulase, resulting in a heightened release of phenolics, flavonoids, and terpenoids into the NADES [50]. As Singh et al., with the increase in time, the content of fermented wheat bran also increased at the initial 15-minute point [51]. Hence, employing a period of 60 minutes reached the highest production of TFC, TPC, TTC, and TPRC, with corresponding values of 9.94±0.72 mg RE/g, 39.25±0.28 mg GAE/g, 39.08±5.01 mg UE/g, and 3.44±0.07 mg BSAE/g.

### 3.5. Comparison of UAE, MAE, EAE, UMAE, UEAE, MEAE, UMEAE techniques

The extraction performance of seven novel extraction techniques is compared via bioactive component contents to find the most suitable extraction method for recovering these compounds from DBMP. Table 2 shows the antioxidant capacity, total phenolic, flavonoid, terpenoid, and protein contents of DBMP extracts obtained at the proper conditions of UAE, MAE, and UMAE. NADES-based UAE exhibited significantly higher extraction efficiency for TPC, TFC, and TTC compared to NADES-based MAE and NADES-based EAE. In terms of supporting the extraction of bioactive compounds, the extraction efficiency followed the order UAE > MAE > EAE, whereas the extraction of protein content (TPRC) showed a different trend, with MAE > UAE > EAE. However, the disadvantages of single methods include the generation of free radicals during ultrasonic treatment, the overheating of microwave treatment, and low mass transfer during enzyme treatment. Therefore, combining the novel extraction techniques to address their drawbacks is necessary. The detailed experimental conditions of combined methods are presented in S1 Table.

**Table 2:** The results of NADES-based UAE, MAE, EAE, UMAE, UEAE, MEAE and UMEAE.

Criteria/Extraction techniques	NADES-based UAE	NADES-based MAE	NADES-based UMAE
Solvents	L-Gly (molar ratios of 2:1)		
TFC (mg RE/g)	34.41±3.55c	18.65±0.35d	39.55±2.41b
TPC (mgGAE/g)	70.73±2.18d	51.9±1.19e	77.61±2.63b
TTC (mg UE/g)	75.53±2.1d	48.86±4.74e	86.92±1.41b
TPRC (mg BSAE/g)	10.477±0.69b	14.9±0.08a	7.03±0.23d
ABTS (mMTE/gdw)	6.48±0.07d	6±0.24d	8.66±0.16b
DPPH (mMTE/gdw)	8.2±0.04cd	7.51±0.48d	9.84±0.58b
OH (mMTE/gdw)	14.97±1.56d	12.22±1.37d	19.75±0.38d

Criteria/Extraction techniques	NADES-based EAE	NADES-based UEAE	NADES-based MEAE	NADES-based UMEAE
Solvents	L-Gly-Na (molar ratios of 2:4:1)			
TFC (mg RE/g)	9.94±0.72e	38.17±0.56b	37.38±0.5bc	45.15±1.67a
TPC (mgGAE/g)	39.25±0.28f	73.98±1.15c	72.75±2.33cd	95.52±1.36a
TTC (mg UE/g)	39.08±5.01f	82.4±1.05bc	80.13±1.29cd	105.24±2.28a
TPRC (mg BSAE/g)	3.44±0.07e	8.18±0.77c	8.93±1.12c	6.57±0.1d
ABTS (mMTE/gdw)	5.02±0.29e	8.45±0.46b	7.54±0.4c	10.72±0.73a
DPPH (mMTE/gdw)	4.7±0.62e	9.35±0.15bc	8.62±0.6bcd	12.87±1.92a
OH (mMTE/gdw)	9.44±1.18d	508.47±34.63b	293.45±26.68c	633.83±17.57a

Note: Different characters (a, b, c, d, e, and f) show the significance of statistical analysis

The findings indicated that TPC, TFC, and TTC extracted using NADES-based UMAE exceeded those obtained through UEAE and MEAE. This finding can be ascribed to the cooperation of the overheating effect from microwaves and the acoustic cavitation effect from ultrasound on the plant matrix, enhancing the diffusion of terpenoids and phenolics from cytosol into solvents. The introduction of ultrasound induces the formation of numerous microchannels on the material’s surface, which can facilitate heat and mass transfer during MAE. Moreover, the elevated temperature produced through microwaves diminishes viscosity and prompts cell rupture, thereby amplifying the liberation of terpenoids, flavonoids, and phenolics. The low extraction efficiency of NADES-based UEAE and MEAE can be attributed to the less destructive effect of Cellulast 1.5L on the plant cell walls of DBMP than ultrasound and microwave.

When comparing NADES-based UAE, UMAE, and UMEAE, it can be understood that combining various treatments can further disrupt the cell membrane, increasing the release of additional bioactive compounds. Therefore, it is evident that the content of bioactive compounds in NADES-based UMEAE > UMAE > UAE. In terms of the extraction efficiency of TPRC in combined extraction methods, the results show an opposite trend to the extraction efficiency of bioactive compounds: NADES-based MEAE > UEAE > UMAE > UMEAE. This finding is attributed to the salting-out phenomenon, in which small-sized bioactive molecules (phenolic, flavonoid, terpenoid) compete for hydrogen bonding with NADES solvent, making proteins less soluble [52]. Regarding antioxidant activity, the extract of NADES-based UMEAE exhibits the highest activity, followed by the extract of NADES-based UMAE, and the extract with the lowest antioxidant activity is NADES-based EAE. This phenomenon can be accounted for by the higher phenolic, flavonoid, and terpenoid content, which possess higher antioxidant activity [53]. Our previous study also reported that using combined methods showed more extraction efficiency than single methods in recovering bioactive compounds from *Abelmoschus sagittifolius* (Kurz) Merr roots. Therefore, the more efficient the extraction method for bioactive compounds, the higher the antioxidant activity of the extract.

### 3.6. Surface morphology variation of DBMP

**Fig 4:**
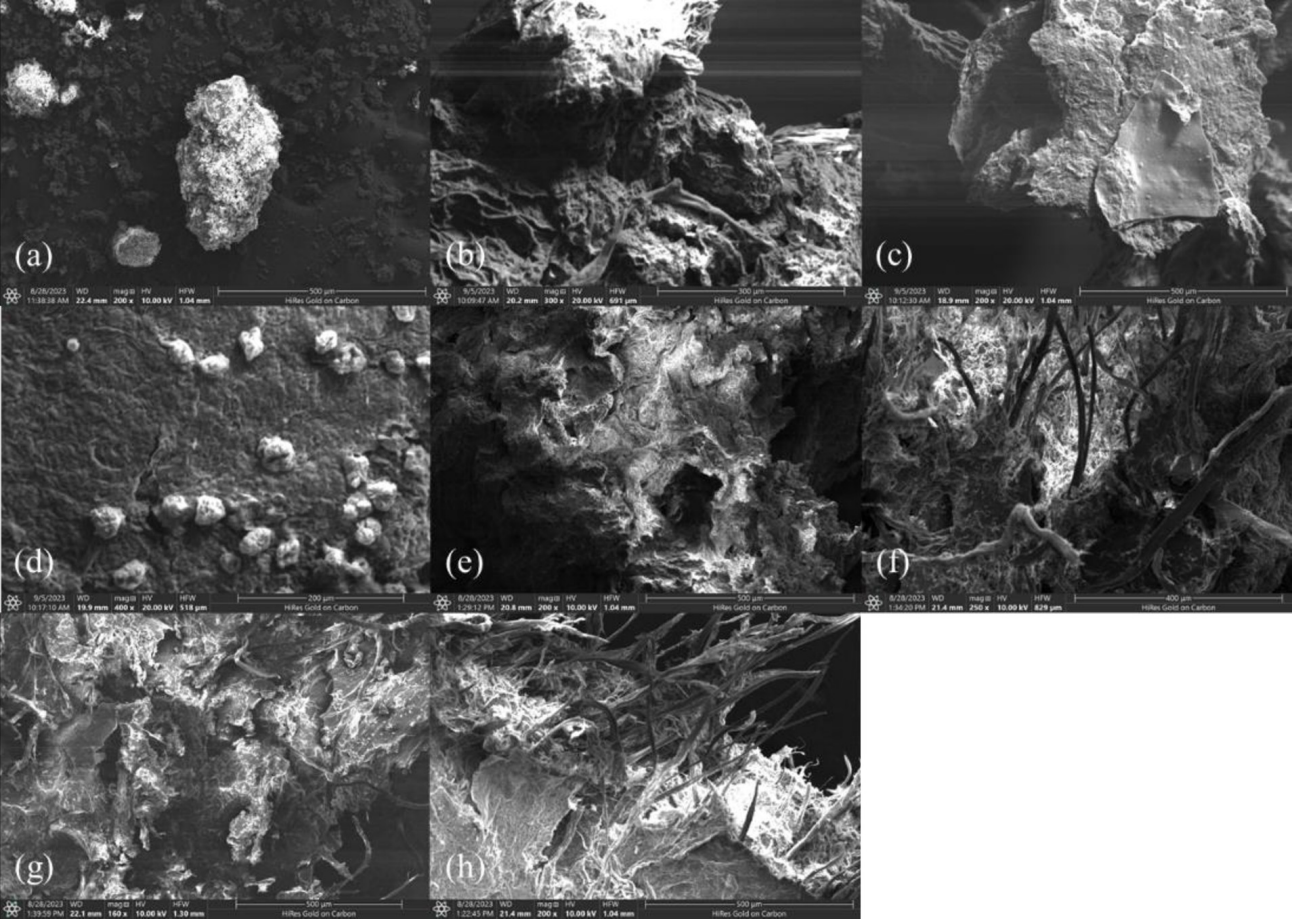
The variation in the surface of material with and without treatment; (a): material without treatment; (b): material with NADES-based UAE treatment; (c): material with NADES-based MAE treatment; (d): material with NADES-based EAE treatment; (e): material with NADES-based UMAE treatment; (f): material with NADES-based UEAE treatment; (g): material with NADES-based MEAE treatment; (h): material with NADES-based UMEAE treatment.

Scanning electron microscopy (SEM) is commonly employed to observe and collect data about plant cells’ surface morphology and physiological state before and after treatment [54]. Therefore, SEM was employed to explore the surface structure of DBMP following its exposure to seven distinct methods (UAE, MAE, EAE, UMAE, UEAE, MEAE, and UMEAE). After undergoing treatments, the extent of cell wall damage exhibited the following order: UMEAE induced the most significant destruction, followed by UEAE and UMAE (which showed similar levels of destruction as depicted in Fig 4A–4H. In contrast to the integrity surface of untreated DBMP, the external appearance of DBMP subjected to UMEAE treatment exhibited significant structural deterioration, characterized by the presence of extensive pores and fractures, as illustrated in Fig 4A–4H. This observable damage can be attributed to the combination of NADES, the acoustic cavitation effect, the heating effect of microwave, and enzymatic hydrolysis, resulting in the significant degradation of the DBMP surface. These results agreed with Manel Elakremi et al., who used MAE to recover bioactive compounds from Pistacia vera L. leaves [54]. The same trend was also observed by our previous study, which investigated the impact of NADES-based novel extraction techniques on spent tea leaves and *Abelmoschus sagittifolius* (Kurz) Merr roots [5, 47] These findings affirm that UMEAE was shown as an effective technique for destroying plant cell walls.

## 4. Conclusion

In this research, L-Gly was highly efficient in producing TPC, TFC, TTC, and TPRC from DBMP. The high extraction efficiency of L-Gly stems from the creation of hydrogen bond networks between solvents and target analytes. The study also examined the effect of extraction conditions, such as LSR, water content, temperature, enzyme concentration, microwave, and ultrasonic power on the recovery yield of bioactive compounds. The applicable conditions for NADES-based UAE extracting bioactive compounds and proteins were 50 mL/g LSR, with 30% water content in L-Gly, 50°C, and 600 W ultrasonic power in 15 min. As regards NADES-based MAE, the suitable conditions for extracting bioactive compounds and proteins were 40 mL/g LSR, 30% water content in L-Gly, and 400 W microwave power in 3 min. As for NADES-based EAE, the appropriate conditions for bioactive compounds and protein extraction were 30 mL/g LSR and an enzyme concentration of 20 U/g in L-Gly-Na molar ratio at 2:4:1, 40% water content in 60 min. EAE results demonstrated that NADES acts as a medium for enzymatic-induced hydrolysis to degrade the cell walls. Additionally, NADES can synergize with novel extraction techniques to foster the extraction yield. NADES-based UMEAE, which combined green solvents, UAE, MAE, and EAE, presented the highest level of antioxidant activity, followed by NADES-based UMAE. Meanwhile, NADES-based EAE exhibited the lowest antioxidant activity. Additionally, SEM images revealed that NADES-based UMEAE had the most destructive impact on the DBMP surface compared to other extraction techniques. Moreover, the antioxidant activities of bioactive compounds were measured. NADES-based UMEAE showed the highest antioxidant activities due to the conversion of bound phenolics to free ones via the hydrolysis linkages between polysaccharides and phenolics. These findings support the idea that DBMP extracts are a potential source of bioactive compounds in food products. The utilization of NADES-based UMAE offers a green, sustainable, and effective method for the recovery of bioactive compounds and proteins from DBMP.

## Supporting information

S1 Table The extraction conditions and results of NADES-based UAE, MAE, EAE, UMAE, UEAE, MEAE and UMEAE. (DOCX)

S1 Graphical abstract (TIF)
