# Supplementary material for: Extracting bioactive compounds and proteins from Bacopa monnieri using natural deep eutectic solvents
Source: PLoS One. 2024 Mar 29;19(3):e0300969. doi: 10.1371/journal.pone.0300969 (PMC10980249; doi:10.1371/journal.pone.0300969)
Supplement: S1 Table — (DOCX) [file pone.0300969.s001.docx]

S1 Table The extraction conditions and results of NADES-based UAE, MAE, EAE, UMAE, UEAE, MEAE and UMEAE

| **Criteria/Extraction techniques** | **NADES-based UAE** | **NADES-based MAE** | **NADES-based UMAE** |
| --- | --- | --- | --- |
| Solvents | L-Gly (molar ratios of 2:1) | | |
| Conditions |  |  |  |
| LSR (mL/g) | 50 | 40 | 40 |
| Water content (%) | 30 | 30 | 30 |
| Ultrasonic Temperature (°C) | 50 |  | 50 |
| Ultrasonic power (W) | 600 |  | 600 |
| Sonication time (min) | 15 |  | 15 |
| Microwave power (W) |  | 400 | 400 |
| Microwave irradiation time (min) |  | 3 | 3 |
| TFC (mg RE/g) | 34.41±3.55c | 18.65±0.35d | 39.55±2.41b |
| TPC (mg GAE/g) | 70.73±2.18d | 51.9±1.19e | 77.61±2.63b |
| TTC (mg UE/g) | 75.53±2.1d | 48.86±4.74e | 86.92±1.41b |
| TPRC (mg BSAE/g) | 10.477±0.69b | 14.9±0.08a | 7.03±0.23d |
| ABTS (mM TE/g dw) | 6.48±0.07d | 6±0.24d | 8.66±0.16b |
| DPPH (mM TE/g dw) | 8.2±0.04cd | 7.51±0.48d | 9.84±0.58b |
| OH (mM TE/g dw) | 14.97±1.56d | 12.22±1.37d | 19.75±0.38d |

| **Criteria/Extraction techniques** | **NADES-based EAE** | **NADES-based UEAE** | **NADES-based MEAE** | **NADES-based UMEAE** |
| --- | --- | --- | --- | --- |
| Solvents | L-Gly-SC (2:4:1) | | | |
| Conditions |  |  |  |  |
| LSR (mL/g) | 30 | 30 | 30 | 30 |
| Enzyme Concentration (U/g) | 20 | 20 | 20 | 20 |
| Molar ratios of glycerol | 4 | 4 | 4 | 4 |
| Water content (%) | 40 | 40 | 40 | 40 |
| Hydrolysis time (min) | 60 | 60 | 60 | 60 |
| Ultrasonic Temperature (°C) |  | 50 |  | 50 |
| Ultrasonic power (W) |  | 600 |  | 600 |
| Sonication time (min) |  | 15 |  | 15 |
| Microwave power (W) |  |  | 400 | 400 |
| Microwave irradiation time (min) |  |  | 3 | 3 |
| TFC (mg RE/g) | 9.94±0.72e | 38.17±0.56b | 37.38±0.5bc | 45.15±1.67a |
| TPC (mg GAE/g) | 39.25±0.28f | 73.98±1.15c | 72.75±2.33cd | 95.52±1.36a |
| TTC (mg UE/g) | 39.08±5.01f | 82.4±1.05bc | 80.13±1.29cd | 105.24±2.28a |
| TPRC (mg BSAE/g) | 3.44±0.07e | 8.18±0.77c | 8.93±1.12c | 6.57±0.1d |
| ABTS (mM TE/g dw) | 5.02±0.29e | 8.45±0.46b | 7.54±0.4c | 10.72±0.73a |
| DPPH (mM TE/g dw) | 4.7±0.62e | 9.35±0.15bc | 8.62±0.6bcd | 12.87±1.92a |
| OH (mM TE/g dw) | 9.44±1.18d | 508.47±34.63b | 293.45±26.68c | 633.83±17.57a |
