# Supplementary figures and images for: Extracting bioactive compounds and proteins from Bacopa monnieri using natural deep eutectic solvents
Source: PLoS One. 2024 Mar 29;19(3):e0300969. doi: 10.1371/journal.pone.0300969 (PMC10980249; doi:10.1371/journal.pone.0300969)

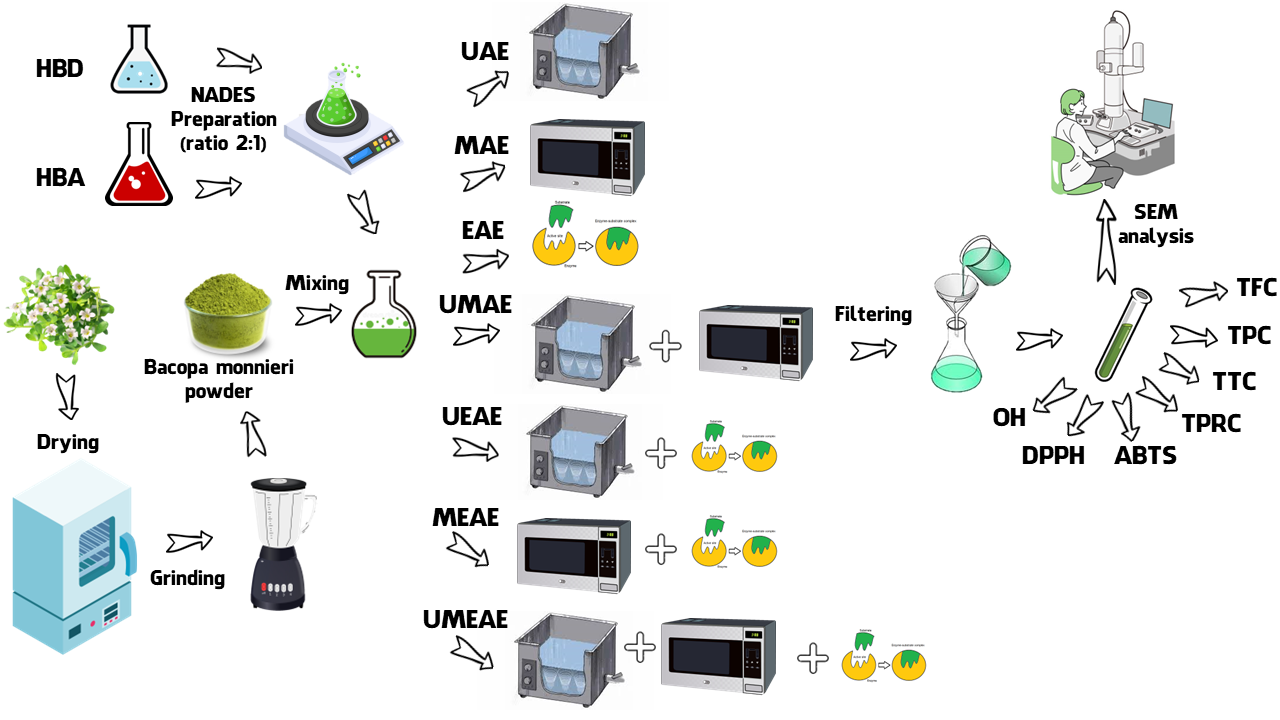

Supplement: S1 Graphical abstract — (TIF) [file pone.0300969.s002.tif]
